# Diagnostic Value of the Methylation of Multiple Gene Promoters in Serum in Hepatitis B Virus-Related Hepatocellular Carcinoma

**DOI:** 10.1155/2017/2929381

**Published:** 2017-08-29

**Authors:** Xueyan Dong, Qiang Hou, Yueming Chen, Xianjun Wang

**Affiliations:** ^1^Department of Laboratory Medicine, Hangzhou First People's Hospital, Hangzhou, Zhejiang 310006, China; ^2^Hangzhou Cancer Institution, Hangzhou Cancer Hospital, Hangzhou, Zhejiang 310002, China

## Abstract

This study sought to evaluate the diagnostic value of the methylation of multiple gene promoters in serum in hepatitis B virus- (HBV-) related hepatocellular carcinoma (HCC). A total of 343 participants were enrolled, including 98 patients with HCC, 75 patients with liver cirrhosis (LC), 90 patients with chronic hepatitis B (CHB), and 80 healthy individuals. *RASSF1A*, *APC*, *BVES*, *TIMP3*, *GSTP1*, and *HOXA9* were selected as the candidate genes. The MethyLight method was used to assay promoter methylation statuses. The diagnostic performances of markers were assessed by constructing receiver operating characteristic (ROC) curves. The prevalences of methylation for *RASSF1A*, *APC*, *BVES*, *HOXA9*, *GSTP1*, and *TIMP3* were 52.04%, 36.73%, 29.59%, 20.41%, 17.35%, and 11.22%, respectively. *APC* methylation completely overlapped with *RASSF1A* methylation. The area under the curve (AUC) for *RASSF1A* methylation (0.718) was better than the corresponding AUC for AFP (0.609) in distinguishing HCC from CHB. When *RASSF1A*, *BVES*, *HOXA9*, and AFP were combined, the AUC was 0.852 (95% CI = 0.796–0.908, *P* = 0.028), and the sensitivity and specificity were 83.7% and 78.9%, respectively. In conclusion, an assay that combines methylation of the *RASSF1A*, *BVES*, and *HOXA9* gene promoters in serum and AFP could significantly improve HBV-related HCC diagnoses.

## 1. Introduction

Hepatocellular carcinoma (HCC) is one of the most common human malignant tumors and causes an estimated 50 million deaths per year worldwide [1, 2]. Chronic hepatitis B virus (HBV) infection is a major cause of HCC in East Asia. Currently, a serum alpha fetoprotein (AFP) assay is commonly used to detect HCC. However, this assay has relatively low sensitivity and specificity; therefore, its clinical application is limited. Given current clinical diagnostic tools, patients with HCC may not undergo effective treatment. Thus, most such patients have already progressed to an advanced disease stage by the time they are definitively diagnosed. Therefore, there is an urgent need to identify effective biomarkers for HCC.

CpG island methylation in the gene promoter is an important epigenetic mechanism that is often involved in carcinogenesis. Certain tumor suppressor genes and other pivotal genes that regulate cell signaling pathways are frequently silenced in tumor tissues due to promoter hypermethylation [3–5]. These epigenetic changes have been tested and shown to be potential markers for HCC [6]. However, it is inconvenient and invasive to use tumor tissues to detect promoter methylation in high-risk populations. Serum DNA from patients with cancer is derived from apoptotic cells, necrotic cells, or circulating tumor cells and reflects a variety of DNA changes in the forms of allelic imbalance, DNA integration, mutation, and methylation [7–9]. These changes in serum DNA are highly consistent with those present in tumor tissues [10, 11]. Prior literature indicates that the methylation of gene promoters in serum could be a promising noninvasive biomarker for diagnosing HCC.

In this study, RAS association domain family protein 1A (*RASSF1A*), adenomatous polyposis coli (*APC*), blood vessel epicardial substance (*BVES*), TIMP metallopeptidase inhibitor 3 (*TIMP3*), glutathione S-transferase pi 1 (*GSTP1*), and homeobox A9 (*HOXA9*) were selected as candidate targets; these genes are frequently methylated during carcinogenesis of digestive tract carcinoma [12–15]. The MethyLight method [16] was used to measure serum methylation statuses of genes in patients with HCC, patients with liver cirrhosis (LC), patients with chronic hepatitis B (CHB), and healthy subjects. Subsequently, the diagnostic performances of the selected markers were assessed by constructing receiver operating characteristic (ROC) curves.

## 2. Materials and Methods

### 2.1. Study Population

A total of 343 participants who visited Hangzhou First People's Hospital from January 2011 to December 2015 were enrolled in the study. They were divided into four age- and gender-matched groups (HCC patients, LC patients, CHB patients, and healthy subjects). In detail, the 98 patients with HCC had been diagnosed via liver ultrasound, computed tomography (CT), serum AFP level, and ultimately histological examination. The 75 patients with LC had been diagnosed via liver ultrasound and CT and exhibited LC accompanied by portal hypertension and hypersplenism. The 90 patients with CHB satisfied diagnostic criteria based on guidelines for the prevention and treatment of chronic hepatitis B (2010 version) issued by the Chinese Society of Hepatology and the Chinese Society of Infectious Diseases of the Chinese Medical Association. Additionally, the HCC patients, LC patients, and CHB patients had HBV surface antigen- (HBsAg-) positive serum. Subjects who presented with other liver diseases, such as autoimmune hepatitis, alcoholic hepatitis, and infection with another type of hepatitis virus, were excluded from the study. Eighty healthy individuals were obtained from the Physical Examination Center of Hangzhou First People's Hospital. All subjects provided written informed consent, and this study was approved by the ethics committee of Hangzhou First People's Hospital.

### 2.2. Serum DNA Extraction and Sodium Bisulfite Treatment

Five-milliliter samples of peripheral blood were drawn from the patients and healthy subjects. Samples were centrifuged at 2000 ×g for 10 min. Subsequently, 2 ml of serum was collected from each sample via centrifugation at 12000 ×g for 5 min and stored at −80°C until use. A serum DNA extraction kit (GenMagBio Biotechnology Co. Ltd., Beijing, China) was used to extract DNA from 600 *μ*l of serum. Serum DNA was modified via sodium bisulfite treatment and purified using the EpiTect Bisulfite Kit (Qiagen, Hilden, Germany). The aforementioned operations were performed in accordance with the protocols recommended by the manufacturer.

### 2.3. Preparation of Positive Control

One reaction was performed for *in vitro* methylation. The 20 *μ*l reaction system consisted of 2 *μ*l of 10 × NEBuffer, 1 *μ*l of genomic DNA (15 *μ*g/l) from umbilical cord blood from a healthy fetus, 2 *μ*l of S-adenosylmethionine (SAM) (1600 *μ*M), 1 *μ*l of CpG methyltransferase (M.SssI) (4 U/*μ*l) (NEB, Herts, UK), and 14 *μ*l of nuclease-free water. This mixture was incubated at 37°C for 1 h, and the reaction was then stopped at 65°C for 20 min. Methylated genomic DNA was treated and purified using the sodium bisulfite modification approach described above.

### 2.4. DNA Methylation Assay

The methylation status of each gene was examined using methylation-specific quantitative PCR (MethyLight). The sequences of the primers and probes for MethyLight were previously described [17–22]; these primers and probes were synthesized by Shanghai HuiRui Biotechnology Co. Ltd. (Table 1). In this study, actin beta (*ACTB*) was used as the internal reference gene to correct for differences in DNA template quantities among samples. The PCR mixture had a final volume of 20 *μ*l and contained 1 *μ*l of bisulfite-treated DNA, 0.15 *μ*l of each primer (10 *μ*M), 0.1 *μ*l of each probe (10 *μ*M), 9.6 *μ*l of nuclease-free water, and 10.0 *μ*l of 2 × PCR Buffer (Toyobo Co. Ltd., Japan), which consisted of Taq DNA polymerase, reaction buffer, and a deoxynucleotide triphosphate mixture. PCR was performed using an ABI 7500 Sequence Detection System (Life Technologies, USA). The PCR program included an initial denaturation step at 95°C for 3 min followed by 45 cycles of denaturation at 95°C for 10 s and annealing at 60°C for 1 min. M.SssI-treated DNA, normal lymphocyte DNA, and nuclease-free water were used as a positive control, a negative control, and a blank control, respectively. Each sample was assessed in duplicate, with the average of the two duplicates used for analysis. Gene promoter methylation statuses are presented as percentage of methylated reference (PMR) values [23]. A PMR ≥ 4% was classified as positive, whereas a PMR < 4% was classified as negative; this threshold has been validated in the literature as the standard cut-off value for PMR [24–26].

### 2.5. Statistical Analysis

The Mann–Whitney *U* test was used to examine differences in nonparametric variables. Associations between methylation and clinicopathologic parameters were determined using chi-square (*χ*^2^) tests. Diagnostic efficacies were presented as areas under ROC curves (AUCs). *P* values <0.05 were regarded as statistically significant. All data analyses were performed using SPSS software, version 21 (IBM, Armonk, NY, USA).

## 3. Results

### 3.1. Demographic Characteristics

General clinical information was collected for 343 subjects. Serum levels of alanine aminotransferase (ALT), albumin (ALB), total bilirubin (TBIL), AFP, and blood platelet (PLT) count significantly differed among the four groups (all *P* < 0.05). In particular, relative to the healthy control group, the HCC, LC, and CHB groups had higher ALT, TBIL, and AFP levels but lower ALB and PLT levels (all *P* < 0.05).

### 3.2. Serum Methylation Statuses of Multiple Gene Promoters

Rates of hypermethylation of *RASSF1A*, *APC*, *BVES*, *TIMP3*, *GSTP1*, and *HOXA9* promoters in HCC patients, LC patients, CHB patients, and healthy individuals are shown in Table 2. In HCC patients, the prevalences of hypermethylation for *RASSF1A*, *APC*, *BVES*, *HOXA9*, *GSTP1*, and *TIMP3* were 52.04%, 36.73%, 29.59%, 20.41%, 17.35%, and 11.22%, respectively, and *APC* methylation completely overlapped with *RASSF1A* methylation. In addition, *RASSF1A* methylation was sometimes detected in serum from LC patients (13.33%) but infrequently observed in serum from healthy subjects (3.75%). The other 5 genes showed low methylation rates in LC patients (2.67%–5.33%) and no detected methylation in healthy subjects.

### 3.3. Powers of Methylation Statuses of Multiple Genes and the AFP Assay to Distinguish HCC from CHB

For discriminating between HCC and CHB, the sensitivity of *RASSF1A* methylation in serum was greater than the sensitivities of the other diagnostic indicators, whereas the sensitivities of *BVES*, *APC*, *TIMP3*, *GSTP1*, and *HOXA9* methylation in serum were all lower than the sensitivity of AFP (≥20 ng/l) (Table 3). For all 6 genes, the specificities of promoter methylation in serum were better than the specificity of AFP (≥20 ng/l). Because *APC* methylation completely overlapped with *RASSF1A* methylation, the AUCs of serum *RASSF1A* methylation (0.718), *BVES* methylation (0.636), AFP (≥20 ng/l) (0.609), and *HOXA9* methylation (0.521) indicated that these metrics were the top 4 indicators for distinguishing between HCC and CHB.

### 3.4. Powers of Combined Assays to Distinguish HCC from CHB

**Figure 1:**
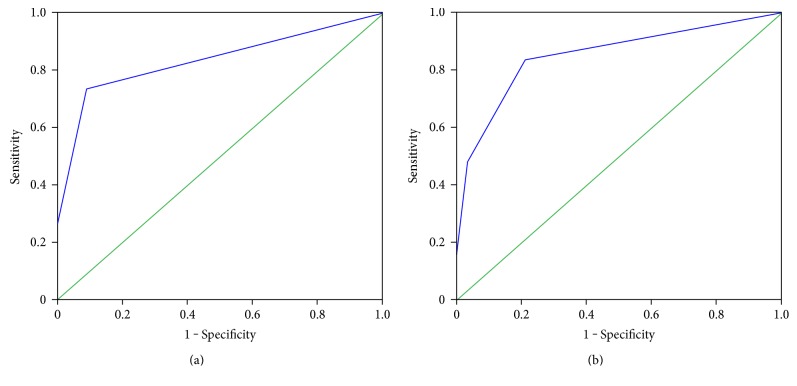
Receiver operating characteristic (ROC) curves analyzing the use of *RASSF1A*, *BVES*, and *HOXA9* methylation in serum and AFP (≥20 ng/l) for distinguishing hepatocellular carcinoma (HCC) from chronic hepatitis B (CHB). (a) When *RASSF1A*, *BVES*, and *HOXA9* methylation statuses in serum were utilized together, the AUC was 0.834 (95% CI = 0.774–0.894, *P* = 0.031), the sensitivity was 73.5%, and the specificity was 91.1%. (b) When *RASSF1A*, *BVES*, and *HOXA9* methylation statuses in serum and AFP were combined, the AUC was 0.852 (95% CI = 0.796–0.908, *P* = 0.028) and the sensitivity and specificity were 83.7% and 78.9%, respectively.

To further investigate the diagnostic value of combining methylation statuses of *RASSF1A*, *BVES*, and *HOXA9* in serum and AFP (≥20 ng/l) to distinguish HCC from CHB, ROC curves were constructed. The results showed that when methylation statuses of *RASSF1A*, *BVES*, and *HOXA9* in serum were utilized together, the AUC was 0.834 (95% CI = 0.774–0.894, *P* = 0.031) and the sensitivity and specificity were 73.5% and 91.1%, respectively. For a combination of the methylation statuses of *RASSF1A*, *BVES*, and *HOXA9* in serum and AFP (≥20 ng/l), the AUC was 0.852 (95% CI = 0.796–0.908, *P* = 0.028) and the sensitivity and specificity were 83.7% and 78.9%, respectively (Figure 1).

## 4. Discussion

In areas where HBV infection is highly prevalent, a chronic liver disease spectrum has been formed that extends from CHB to LC and even to progression to HCC [1, 2]. Therefore, having patients with chronic HBV infection undergo regular assessments using a powerful indicator would help improve HCC diagnoses and the timeliness of treatment. Aberrant gene promoter hypermethylation has been proposed as an approach for diagnosing solid tumors. Specifically, an assay of DNA methylation in serum could be a noninvasive method for assessing a reliable biomarker for tumors.

In this study, a magnetic bead method was used to isolate and purify serum DNA, and the MethyLight method was used to perform the DNA methylation assay. These methods guaranteed the reliability of the study results. Six tumor-associated genes (*RASSF1A*, *APC*, *BVES*, *TIMP3*, *GSTP1*, and *HOXA9*) were selected as candidates. These genes are involved in a variety of cellular functions and signaling pathways, such as cell proliferation, invasion and adhesion (*RASSF1A*, *APC*, and *BVES*), metastasis and angiogenesis (*TIMP*3), detoxification (*GSTP1*), and cell differentiation (*HOXA9*) [12–15]. We found that the highest rate of methylation was observed for *RASSF1A* (52.04%, 51/98), followed by *APC* (36.73%, 36/98), and that *APC* methylation completely overlapped with *RASSF1A* methylation. Notably, methylated *RASSF1A* and methylated *APC* were both sometimes detected in LC and CHB. These results imply that methylation of *RASSF1A* and *APC* may be a common aberrant epigenetic change during the development of HCC and could even be involved in early stages of hepatocarcinogenesis.

Comparisons indicated that *RASSF1A* methylation, *BVES* methylation, AFP (≥20 ng/l) and *HOXA9* methylation were the top 4 biomarkers for distinguishing HCC from CHB but that only *RASSF1A* methylation exhibited better sensitivity (52.0%) and specificity (91.5%) than AFP (48.0% and 73.9%, resp.). Cell-free methylated *RASSF1A* exhibited large discrepancies with respect to diagnostic performance, including wide ranges for sensitivity (0.27 to 0.94) and specificity (0.38 to 0.95); this phenomenon was likely due to HCC heterogeneity and the selected methylation assay method [27]. Furthermore, we tested the efficacy of combined assays. A combined assay that included the methylation statuses of *RASSF1A*, *BVES*, and *HOXA9* in serum and AFP (≥20 ng/l) exhibited an improved AUC (0.852), sensitivity (83.7%), and specificity (78.9%). Recently, Lu et al. [28] screened 4 hypermethylated genes (*APC*, *COX2*, *RASSF1A*, and *miR-203*) for diagnosing HBV-related HCC using a high-throughput approach. In that study, the 4 biomarkers were combined to form a plasma methylation predictive panel that achieved a sensitivity of 84.2%, a specificity of 83.0%, and an AUC of 0.87 with respect to discriminating between HBV-related HCC and noncancerous control samples. These data illuminated the high diagnostic potential of methylated markers in cell-free DNA from HCC patients. In addition, several literature reports have demonstrated correlations between clinicopathological characteristics of HCC, including clinical prognosis, and such markers [27–29].

Currently, molecular pathologic epidemiology (MPE) is an emerging field of epidemiology based on molecular classification of cancer [30]. MPE research links between various exposures and molecular pathology. Similarly, it can be expanded with circulating biomarkers. The involvement of HBV infection in epigenetic alternations during hepatocarcinogenesis has been described; HBV X (HBx) protein expression promoted DNA methyltransferase (DNMT) activity by upregulation of DNMT1, DNMT3A1, and DNMT3A2 and selectively facilitated regional hypermethylation of specific tumor suppressor genes [4]. In combination of the results of this study, circulating methylated biomarkers are worthy to identify in HBV-related HCC in the future. On the other hand, one case-control study suggested that a disintegrin and metalloproteinase with thrombospondin motifs 5 (*ADAMTS5*) polymorphism was identified to be a useful marker for aflatoxin B1- (AFB1-) related HCC diagnosis and prognosis [31]. Hence, integrative analysis of various exposures and molecular markers is the fundamental premise of precision medicine for HCC. Fortunately, it is increasingly feasible to apply advanced omics technologies to screen specific cancer datasets; this advancement has provided enormous opportunities for molecular classification, personalized prevention, and therapy for the highly heterogeneous diseases including HCC [32]. Nevertheless, there are some challenges in MPE research especially with respect to selection bias, sample size limitations, measurement error and multidisciplinary research environment, and so forth [30].

## 5. Conclusions

In summary, an assay that combines methylation of the *RASSF1A*, *BVES*, and *HOXA9* gene promoters in serum and AFP could significantly improve HCC diagnoses for patients with chronic HBV infection.

## Figures and Tables

**Table 1:** List of primers and probes for MethyLight.

Gene	Primer and TaqMan probe sequences (5′ → 3′)
*RASSF1A*	Forward primer: GCGTTGAAGTCGGGGTTC
	Reverse primer: CCCGTACTTCGCTAACTTTAAACG
	TaqMan probe: FAM-FAM-ACAAACGCGAACCGAACGAAACCA-BHQ1
*APC*	Forward primer: AGTGCGGGTCGGGAAGC
	Reverse primer: AACCACATATCGATCACGTACG
	TaqMan probe: FAM-AAAACGCCCTAATCCGCATCCAACG-BHQ1
*BVES*	Forward primer: GGACGGAGTGGGCGATATC
	Reverse primer: CCTCGAACCGCGCAAA
	TaqMan probe: FAM-CCTACGTACAACCGAACG-MGB
*TIMP3*	Forward primer: GCGTCGGAGGTTAAGGTTGTT
	Reverse primer: CTCTCCAAAATTACCGTACGCG
	TaqMan probe: FAM-AACTCGCTCGCCCGCCGAA-BHQ1
*GSTP1*	Forward primer: CGTCGTGATTTAGTATTGGGGC
	Reverse primer: CTAATAACGAAAACTACGACGACGAAA
	TaqMan probe: FAM-ATAAGGTTCGGAGGTCGCGAGGTTTTCGT-BHQ1
*HOXA9*	Forward primer: AATAAATTTTATCGTAGAGCGGTAC
	Reverse primer: CATATAACAACTTAATAACACCGAA
	TaqMan probe: FAM-GCGCCCCCATTAACCGTACGCGT-BHQ1
*ACTB*	Forward primer: TGGTGATGGAGGAGGTTTAGTAAGT
	Reverse primer: AACCAATAAAACCTACTCCTCCCTTAA
	TaqMan probe: FAM-ACCACCACCCAACACACAATAACAAACACA-BHQ1

**Table 2:** The positive rates of methylation for promoters of 6 genes in serum [*n* (%)].

Gene	HCC patients (*n* = 98)	LC patients (*n* = 75)	CHB patients (*n* = 90)	Healthy controls (*n* = 80)	*χ* ^2a^	*P* ^a^	*χ* ^2b^	*P* ^b^
*RASSF1A*	51 (52.04)	10 (13.33)	4 (4.44)	3 (3.75)	26.215	0.000	49.078	0.000
*APC*	36 (36.73)	4 (5.33)	2 (2.22)	0 (0)	21.834	0.000	32.543	0.000
*BVES*	29 (29.59)	3 (4.00)	1 (1.11)	0 (0)	16.799	0.000	26.292	0.000
*TIMP3*	11 (11.22)	2 (2.67)	0 (0)	0 (0)	4.477	0.034	10.730	0.001
*GSTP1*	17 (17.35)	2 (2.67)	0 (0)	0 (0)	9.365	0.002	17.164	0.000
*HOXA9*	20 (20.41)	4 (5.33)	3 (3.33)	0 (0)	8.081	0.004	10.863	0.001

HCC: hepatocellular carcinoma; LC: liver cirrhosis; CHB: chronic hepatitis B. ^a^HCC patients versus LC patients, *P* < 0.05. ^b^HCC patients versus CHB patients, *P* < 0.01.

**Table 3:** Powers of the methylation statuses of multiple genes and the AFP assay for distinguishing HCC from CHB.

Indicator	Sensitivity (%)	Specificity (%)	AUC
*RASSF1A*	52.0	91.5	0.718
*APC*	36.7	96.4	0.650
*BVES*	29.6	97.6	0.636
*TIMP3*	11.2	98.8	0.356
*GSTP1*	17.4	98.7	0.486
*HOXA9*	20.4	95.8	0.521
AFP (≥20 ng/l)	48.0	73.9	0.609

HCC: hepatocellular carcinoma; CHB: chronic hepatitis B; AUC: area under the curve.

## References

[1] Fung S. K., Lok A. S. (2005). Management of patients with hepatitis B virus-induced cirrhosis. *Journal of Hepatology*.

[2] Tanaka M., Katayama F., Kato H. (2011). Hepatitis B and C virus infection and hepatocellular carcinoma in China: a review of epidemiology and control measures. *Journal of Epidemiology*.

[3] Tischoff I., Tannapfel A. (2008). DNA methylation in hepatocellular carcinoma. *World Journal of Gastroenterology*.

[4] Park I. Y., Sohn B. H., Yu E. (2007). Aberrant epigenetic modifications in hepatocarcinogenesis induced by hepatitis B virus X protein. *Gastroenterology*.

[5] Lim J. S., Park S. H., Jang K. L. (2012). Hepatitis C virus core protein overcomes stress-induced premature senescence by down-regulating p16 expression via DNA methylation. *Cancer Letters*.

[6] Nagashio R., Aral E., Ojima H., Kosuge T., Kondo Y., Kanai Y. (2011). Carcinogenic risk estimation based on quantification of DNA methylation levels in liver tissue at the precancerous stage. *International Journal of Cancer*.

[7] Leon S. A., Shapiro B., Sklaroff D. M., Yaros M. J. (1977). Free DNA in the serum of cancer patients and the effect of therapy. *Cancer Research*.

[8] Matei D. E., Nephew K. P. (2010). Epigenetic therapies for chemoresensitization of epithelial ovarian cancer. *Gynecologic Oncology*.

[9] Levenson V. V., Melnikov A. A. (2012). DNA methylation as clinically useful biomarkers - light at the end of the tunnel. *Pharmaceuticals*.

[10] Jung K., Fleischhacker M., Rabien A. (2010). Cell-free DNA in the blood as a solid tumor biomarker: a critical appraisal of the literature. *Clinica Chimica Acta*.

[11] Dulaimi E., Hillinck J., Ibanez de Caceres I., Al-Saleem T., Cairns P. (2004). Tumor suppressor gene promoter hypermethylation in serum of breast cancer patients. *Clinical Cancer Research*.

[12] Simmer F., Brinkman A. B., Assenov Y. (2012). Comparative genome-wide DNA methylation analysis of colorectal tumor and matched normal tissues. *Epigenetics*.

[13] Han P., Fu Y., Liu J. (2015). Netrin-1 promotes cell migration and invasion by down-regulation of BVES expression in human hepatocellular carcinoma. *American Journal of Cancer Research*.

[14] Guilleret I., Losi L., Chelbi S. T. (2016). DNA methylation profiling of esophageal adenocarcinoma using methylation ligation-dependent macroarray (MLM). *Biochemical and Biophysical Research Communications*.

[15] Kuo C. C., Lin C. Y., Shih Y. L. (2014). Frequent methylation of HOXA9 gene in tumor tissues and plasma samples from human hepatocellular carcinomas. *Clinical Chemistry and Laboratory Medicine*.

[16] Campan M., Weisenberger D. J., Trinh B., Laird P. W. (2009). MethyLight. *Methods in Molecular Biology*.

[17] Fackler M. J., McVeigh M., Mehrotra J. (2004). Quantitative multiplex methylation-specific PCR assay for the detection of promoter hypermethylation in multiple genes in breast cancer. *Cancer Research*.

[18] Fujita N., Kagara N., Yamamoto N. (2014). Methylated DNA and high total DNA levels in the serum of patients with breast cancer following neoadjuvant chemotherapy are predictive of a poor prognosis. *Oncology Letters*.

[19] Zopf S., Ocker M., Neureiter D. (2012). Inhibition of DNA methyltransferase activity and expression by treatment with the pan-deacetylase inhibitor panobinostat in hepatocellular carcinoma cell lines. *BMC Cancer*.

[20] Feng Q., Hawes S. E., Stern J. E. (2008). DNA methylation in tumor and matched normal tissues from non-small cell lung cancer patients. *Cancer Epidemiology, Biomarkers & Prevention*.

[21] Hayashi M., Wu G., Roh J. L. (2015). Correlation of gene methylation in surgical margin imprints with locoregional recurrence in head and neck squamous cell carcinoma. *Cancer*.

[22] Zhang B., Liu S., Zhang Z. (2014). Analysis of BRAFV600E mutation and DNA methylation improves the diagnostics of thyroid fine needle aspiration biopsies. *Diagnostic Pathology*.

[23] Eads C. A., Danenberg K. D., Kawakami K. (2000). MethyLight: a high-throughput assay to measure DNA methylation. *Nucleic Acids Research*.

[24] Eads C. A., Lord R. V., Wickramasinghe K. (2001). Epigenetic patterns in the progression of esophageal adenocarcinoma. *Cancer Research*.

[25] Ogino S., Kawasaki T., Brahmandam M. (2006). Precision and performance characteristics of bisulfite conversion and real-time PCR (MethyLight) for quantitative DNA methylation analysis. *The Journal of Molecular Diagnostics*.

[26] Coleman W. B., Rivenbark A. G. (2006). Quantitative DNA methylation analysis: the promise of high-throughput epigenomic diagnostic testing in human neoplastic disease. *The Journal of Molecular Diagnostics*.

[27] Zhao Z. H., Fan Y. C., Yang Y., Wang K. (2013). Association between Ras association domain family 1A promoter methylation and hepatocellular carcinoma: a meta-analysis. *World Journal of Gastroenterology*.

[28] Lu C. Y., Chen S. Y., Peng H. L., Kan P. Y., Chang W. C., Yen C. J. (2017). Cell-free methylation markers with diagnostic and prognostic potential in hepatocellular carcinoma. *Oncotarget*.

[29] Huang Z. H., Hu Y., Hua D., Wu Y. Y., Song M. X., Cheng Z. H. (2011). Quantitative analysis of multiple methylated genes in plasma for the diagnosis and prognosis of hepatocellular carcinoma. *Experimental and Molecular Pathology*.

[30] Ogino S., Chan A. T., Fuchs C. S., Giovannucci E. (2011). Molecular pathological epidemiology of colorectal neoplasia: an emerging transdisciplinary and interdisciplinary field. *Gut*.

[31] Huang X. Y., Yao J. G., Huang B. C., Ma Y., Xia Q., Long X. D. (2016). Polymorphisms of a disintegrin and metalloproteinase with thrombospondin motifs 5 and aflatoxin B1-related hepatocellular carcinoma. *Cancer Epidemiology, Biomarkers & Prevention*.

[32] Ogino S., Nishihara R., VanderWeele T. J. (2016). The role of molecular pathological epidemiology in the study of neoplastic and non-neoplastic disease in the era of precision medicine. *Epidemiology*.

